# Long-term dynamics of measles in London: Titrating the impact of wars, the 1918 pandemic, and vaccination

**DOI:** 10.1371/journal.pcbi.1007305

**Published:** 2019-09-12

**Authors:** Alexander D. Becker, Amy Wesolowski, Ottar N. Bjørnstad, Bryan T. Grenfell

**Affiliations:** 1 Department of Ecology and Evolutionary Biology, Princeton, New Jersey, United States of America; 2 Department of Epidemiology, Johns Hopkins Bloomberg School of Public Health, Baltimore, Maryland, United States of America; 3 Center for Infectious Disease Dynamics, Pennsylvania State University, University Park, Pennsylvania, United States of America; 4 Fogarty International Center, National Institutes of Health, Bethesda, Maryland, United States of America; 5 Woodrow Wilson School of Public and International Affairs, Princeton University, Princeton, New Jersey, United States of America; ETH Zurich, SWITZERLAND

## Abstract

A key question in ecology is the relative impact of internal nonlinear dynamics and external perturbations on the long-term trajectories of natural systems. Measles has been analyzed extensively as a paradigm for consumer-resource dynamics due to the oscillatory nature of the host-pathogen life cycle, the abundance of rich data to test theory, and public health relevance. The dynamics of measles in London, in particular, has acted as a prototypical test bed for such analysis using incidence data from the pre-vaccination era (1944–1967). However, during this timeframe there were few external large-scale perturbations, limiting an assessment of the relative impact of internal and extra demographic perturbations to the host population. Here, we extended the previous London analyses to include nearly a century of data that also contains four major demographic changes: the First and Second World Wars, the 1918 influenza pandemic, and the start of a measles mass vaccination program. By combining mortality and incidence data using particle filtering methods, we show that a simple stochastic epidemic model, with minimal historical specifications, can capture the nearly 100 years of dynamics including changes caused by each of the major perturbations. We show that the majority of dynamic changes are explainable by the internal nonlinear dynamics of the system, tuned by demographic changes. In addition, the 1918 influenza pandemic and World War II acted as extra perturbations to this basic epidemic oscillator. Our analysis underlines that long-term ecological and epidemiological dynamics can follow very simple rules, even in a non-stationary population subject to significant perturbations and major secular changes.

## Author summary

The impact of intrinsic versus external drivers of transmission on long-term dynamics is an open question in complex systems studies. In particular, when and where dynamics become chaotic has crucial implications for control efforts. Here, we extended the well-studied London measles data to include nearly a century of novel data (1897–1991) that also contains five major demographic changes: the First and Second World Wars, the wartime evacuation of London, the 1918 influenza pandemic, and the start of a measles mass vaccination program. We found that a simple stochastic epidemic model, with minimal historical specifications, can capture the nearly 100 years of dynamics including changes caused by each of the major perturbations. We further illustrated that the majority of dynamic changes are explainable by the internal nonlinear dynamics of the system, tuned by demographic changes. Notably however, the 1918 influenza pandemic and evacuation acted as external perturbations to this basic epidemic oscillator. Yet, in the wake of these massive shifts, the overall system remained stable (Lyapunov exponent < 0), underlining how long-term ecological and epidemiological dynamics can follow very simple rules, even in a non-stationary population subject to significant perturbations and major secular changes.

## Introduction

Predicting transitions between dynamic attractors is a fundamental question in ecology [1]; in particular, how external forcing impacts intrinsic oscillatory dynamics. Phase transitions in nonlinear systems have been extensively studied theoretically [2]. However, examples of systems undergoing multiple shifts are rare due to the time scale of data typically recorded. One exception is the dynamics of fully-immunizing childhood infectious diseases, which have provided an excellent testbed for confronting theory with data [3–5].

Measles, in particular, lends itself to analysis using simple host-pathogen epidemic models. From the early work examining the role of the World War II evacuation [6,7], to more recent work on the predictability of epidemics, low dimensional chaos, and understanding limit cycles [5,8–10], historic measles notification records have helped illuminate the utility, and intersection, of theory and data. In particular, data from the late 1800s and early 1900s from Copenhagen and New York [11,12] and the 1940s-1960s from England and Wales [8,13,14] have revealed recurrent epidemics whose frequency and amplitude change over long-time scales. Although a wide diversity of dynamical regimes has been observed (e.g., regular annual or biennial cycles [8] and chaos [10]), the underlying clockwork–susceptible depletion by infection or vaccination and replenishment by births followed by cycles of human aggregation resulting in seasonal fluctuations in transmission–is ubiquitous [15]. Although both intrinsic (the natural pathogen life history and demography) and extrinsic (local changes to the contact rate between susceptible and infected individuals) processes can drive changes in periodicity, to date few studies have extensively investigated to what extent the dynamics are driven by the internal clockwork versus large external perturbations, and the resulting impact on the frequency and amplitude of epidemics in the long term. Aside from answering core questions in ecology, predicting the epidemic patterns of measles will be important as transient dynamics become more frequent with the continuing measles eradication effort [16].

Due to the temporal scale of data required, there have been few analyses examining the direct impact of demographic changes on the underlying dynamics beyond a simple shift in birth rates or the effect of vaccination on susceptible recruitment. A larger range of external perturbations than those previously tested are possible, such as population fluctuations impacting the force of infection and local variation in contact rates (as the result of public health responses). One analysis [12] examined a long New York time series finding agreement between the observed non-resonant peaks and the predicted transient periods. However, recent work has found differences in measles dynamics between the US and UK suggesting that these results may not be universal across all settings [5], making London an excellent case study for additional analysis. Although discrete time approximations of the mass-action epidemic model work well for incidence data [17,18], even these data are often limited in temporal span. In contrast, mortality data were systematically collected years before incidence data, providing longer periods covering several major demographic perturbations [19]. Incorporating incidence and mortality data into one model has traditionally presented a steep statistical challenge. However, recent advances in statistical particle filtering algorithms [20] now resolves this problem.

Here, we use a newly-digitized time series of measles in London to explore how epidemic dynamics respond to perturbations. We find remarkable stability in the measles epidemiological clockwork over nearly 100 years, even in the face of significant demographic shifts and external perturbations. Studying these perturbations in tandem will yield novel insight into how nonlinear ecological systems respond to perturbations in the mid- and long- term, as well as how predictable limit cycles can be maintained. This should, for example, allow the study of whether perturbations result in multiple shifts between annual, biennial, or even exotic transients (e.g. [21,22]) via changes in both the recruitment of susceptible individuals as well as contact rates. Despite the observed broad population-level changes in London, we find that the dynamical transitions and transients are well-predicted using a simple stochastic compartmental model, as long as it accounts for two major external perturbations (1918 influenza and WWII). A surprising result of our analysis is that a single seasonal transmission pattern (allowing for deviations during the WWII evacuation of children) predicts the key bifurcations related to demographic changes and accurate out-of-sample predictions. Finally, we show through local Lyapunov exponent analysis that, despite numerous bifurcations and perturbations, the attractor remains relatively stable and highly dissipative, thus accounting for the remarkable predictability of a century of London measles dynamics.

## Results

**Fig 1:**
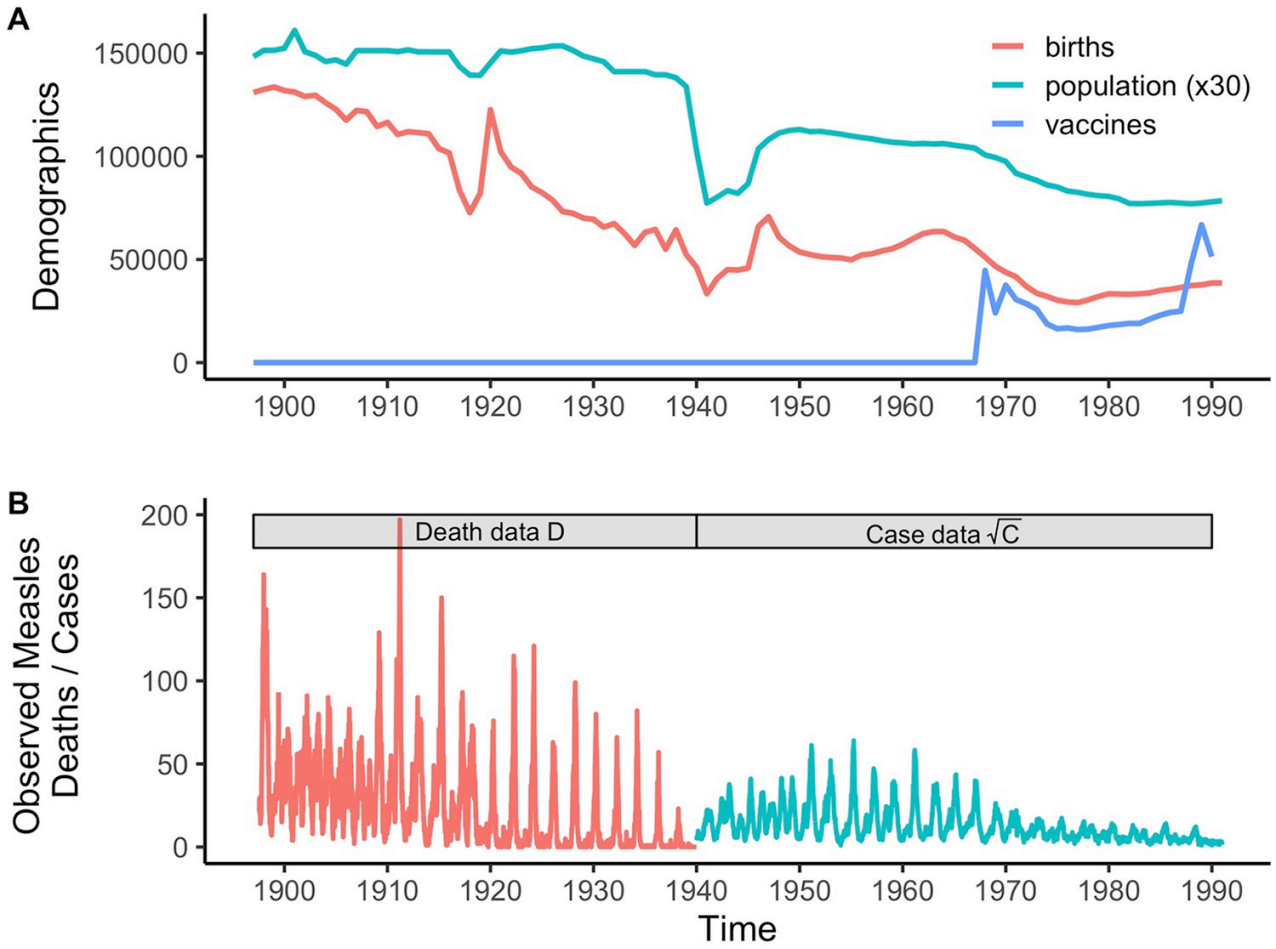
The demographic, vaccination, and measles data analyzed. A) The observed population dynamics shown on a yearly scale. The major demographic fluctuations to births (red) and population counts (green) caused by WWII can be seen starting in 1940. B) Measles dynamics for London 1897–1991, shown on a weekly time scale with mortality (red) until 1940, and incidence (blue) through 1990. Note the case data are shown on a square root scale. Unscaled data are shown (inverted) in Fig 2.

We used particle filtering to fit a seasonally- and demographically-forced SEIR model to combine weekly measles mortality and incidence data reported in London from 1897–1991 (see Fig 1). We quantified the periodicity of the observed dynamics using wavelet analysis. Biennial dynamics predominate (65% of the weeks), although the time series bifurcated from biennial to annual dynamics multiple times (excluding the post-vaccination transients). Although there was a biennial signature around 1910, there is a clear bifurcation from annual to biennial epidemics in 1920. The dynamics remained biennial until returning to an annual attractor in the early 1940s. In 1950, the well-studied post-WWII ‘Baby Boom’ bifurcation occurred, after which dynamics remained firmly biennial until the transient post-vaccination era starting in 1968. Although both World Wars resulted in large shifts in births (Fig 1A), WWII likely had a greater demographic impact due to the war-time evacuation (discussed below).

### Overall dynamics

**Fig 2:**
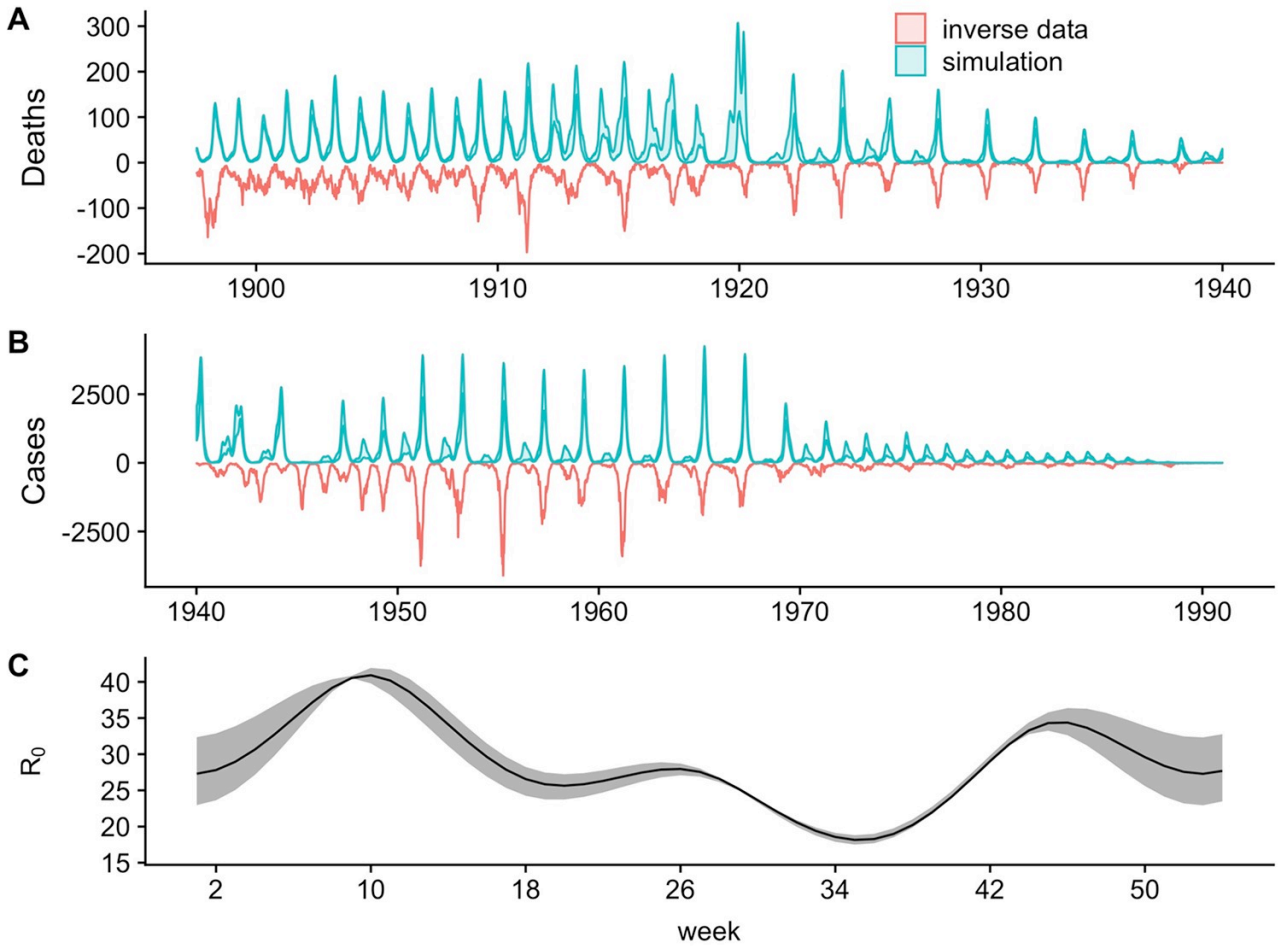
The comparison of predicted against observed weekly measles dynamics for London 1897–1991. A) The 75% quantile fit (blue ribbon) from the forward-simulated fitted model against the inverted death data (red) from 1897 to 1940 while B) shows the fit against the case data from 1940 through 1990. Note that although different data sources are used, the simulation shown here is a fully forward prediction starting in 1897. C) The inferred annual transmission pattern, shown in solid black. The mean yearly transmission rate here is 29. Confidence intervals (95% calculated using the chi-square approximation of the likelihood ratio test) on the inferred seasonality pattern are shown in shaded gray.

**Fig 3:**
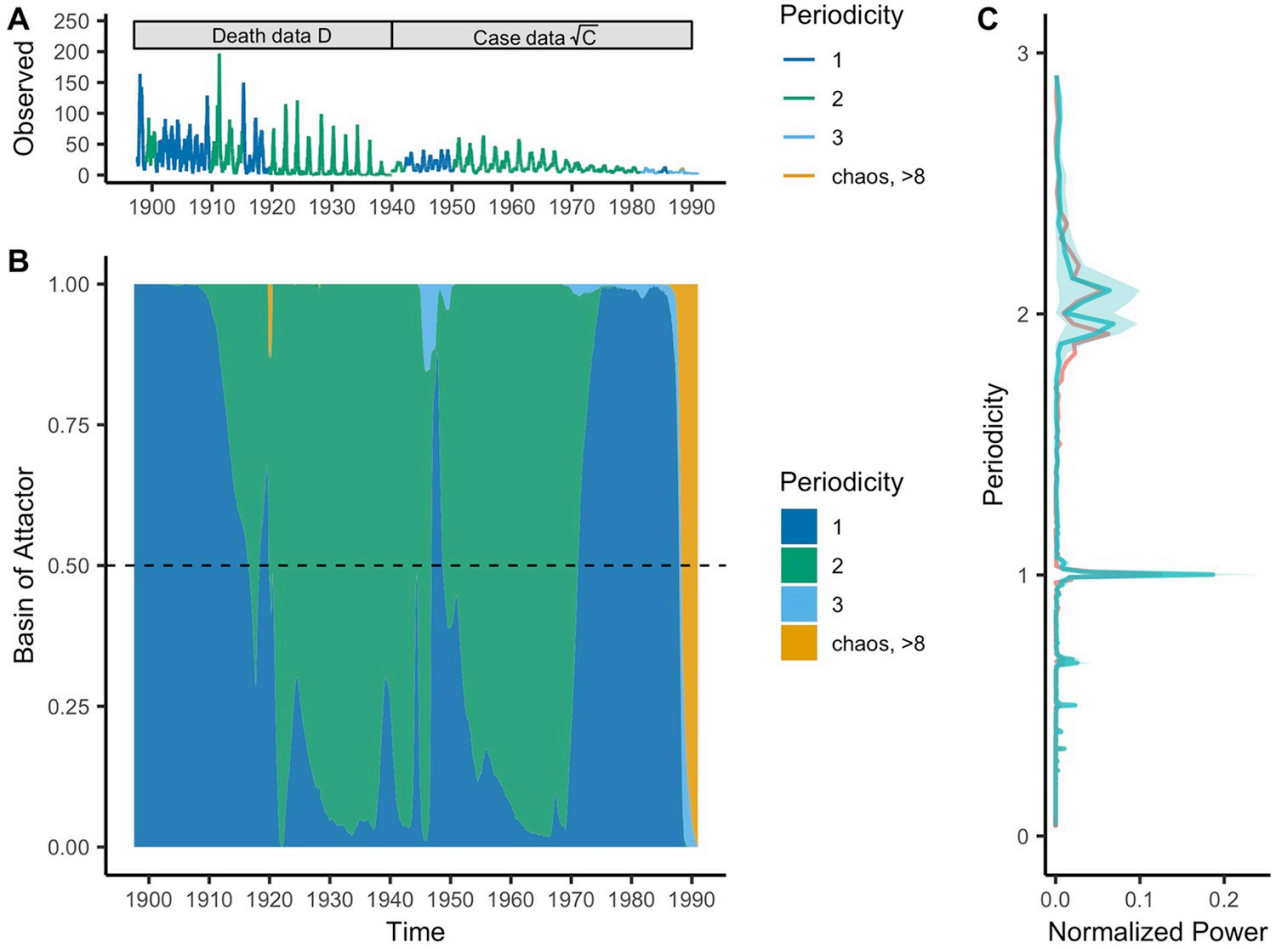
A comparison of predicted against observed measles dynamics for London 1897–1991 based on periodicity inferred from wavelet analysis. A) The observed measles dynamics (death data: 1897–1940, case data: 1940–1991 shown on a square root scale) color coded by the dominant periodicity (in years). B) The density of attractor basins via the simulated stochastic model fitted to the data, also color coded temporally by dominant periodicity. C) The global power spectra of the data (red) against the simulated stochastic model calibrated against the data (blue). Periodicity for all figures is in years (e.g. periodicity 1 in A) and B) refers to annual dynamics). Note, that each simulation shown here is a forward simulation starting from 1897. This figure style is adapted from [12].

We find that, despite numerous bifurcations in the periodicity of epidemics, a single stochastic SEIR model is able to explain the overall behavior of nearly 100 years of limit cycles. We forward simulated the trajectory using the estimated parameters starting from a single set of initial conditions to produce a 4,877-week ahead prediction to test its ability to capture the observed dynamics. Overall, the re-simulated fitted dynamics show remarkably strong agreement with the data using a number of metrics (visual fit of the forward simulation: Fig 2 and power spectra: Fig 3). Using wavelet spectra, we quantified the time-varying periodicity of each forward simulation. Starting in 1897, the SEIR model is firmly annual until 1910, where a slight biennial signature starts to appear in the majority of simulations. The inferred system returns to an annual attractor until 1920, where a strong bifurcation occurs (discussed below), pushing the simulations predominately onto a biennial phase. The trajectory remains closely matched against the data until the WWII evacuation (discussed below). Consistent with previous analyses, the model further predicts the bifurcations seen in the data in 1950 when the baby boom abated. Finally, we see a strong internal dynamic change in 1968 at the start of vaccination. Although, the system overestimated the annual signature compared to the data in this era, both three-year and above signals emerge and finally become dominant as measles is driven to near extinction by immunization.

In order to test the impact of external perturbations on the basic recurrent dynamics, we used the above simulation as a null model purely driven by demographic changes. Variations in demographic rates capture all dynamical shifts except the ones observed in 1920 and 1940 (Fig 2 and S1 Fig). Importantly, demography alone cannot explain the sudden bifurcation observed in 1920 after WWI and the 1918 influenza pandemic. Similarly, demographics alone do not explain the shifts observed during the WWII evacuation.

### External perturbations

To explain discrepancies in the overall forward-simulated trajectory, we incorporated two external perturbations through changes in the contact rates. As hypothesized, our model identified one of the major epidemiological effects of large-scale demographic perturbations to be a change in transmission; we estimate that contact during the 1918 influenza pandemic decreased by 38% (see S1 Fig, parameter estimates shown in S1 Table), a possible side effect of the public health interventions of this time (e.g., partial school closing in response to the pandemic) [23]. Notably, this modulation in contact rate (and not susceptible recruitment alone), is required for the model to predict the empirical bifurcation from annual to biennial around 1920.

**Fig 4:**
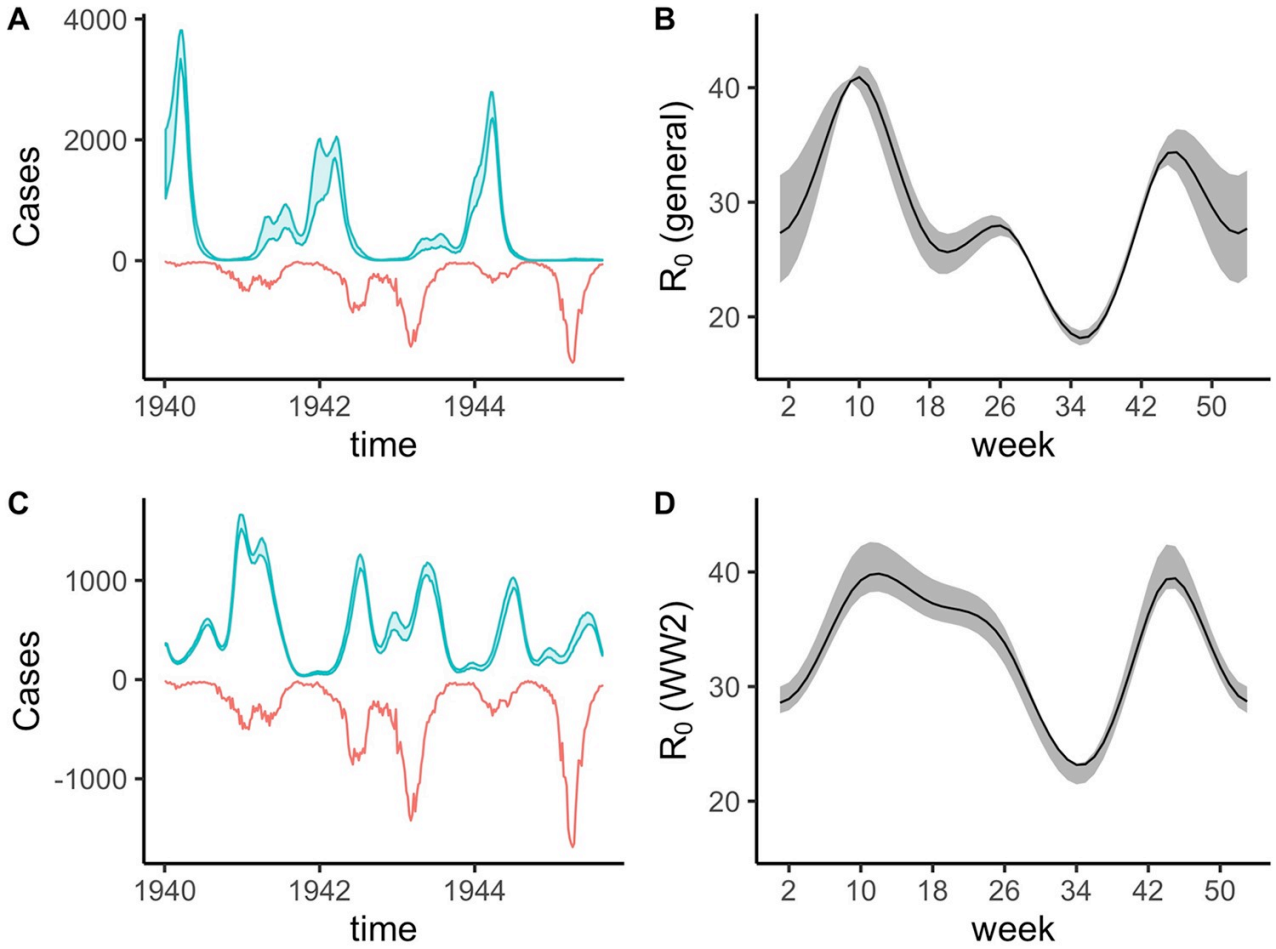
A comparison of predicted against observed measles dynamics for the subsetted WWII time period (1940 to 1946). A) The predicted dynamics using the fitted model against the whole time series with the same visual fit information as Fig 2. B) The inferred seasonality across the whole-time series with mean R_0_ = 29. C) The predicted dynamics fit to just the WWII time period with the inferred seasonality in D). D) The inferred seasonality in just the WWII time period with average R_0_ = 33. Note the local WWII fit produces a lower amplitude seasonality pattern. In both B) and D) 95% confidence intervals (calculated using the chi-square approximation of the likelihood ratio test) on the inferred seasonality pattern are shown in shaded gray, while the inferred values are shown in solid black. Note that the seasonality pattern in D) yields a stronger fit the data while maintaining a generally lower amplitude.

The impacts of WWI did not result in demographic changes large enough to overpower the 1918 effect. The dynamical impacts of WWII, however, were far greater. During the WWII period, the null model fit departs from the data both in outbreak magnitude and periodicity (see Figs 2 and 3). However, unlike the 1918 pandemic, we were unable to identify parameters for the reduction in contact due to WWII. Since this time period includes the wartime evacuation of school-age children from cities, there were likely key differences in seasonality and mixing patterns associated with the major movement of children in the early 1940s. When we fit the model independently to the six-year war epoch of data and use the difference between maximum and minimum seasonality (normalized by the mean) as a measure of amplitude, we find that the WWII time period is the lowest amplitude epoch across the entire time series. This provides evidence of a strong external impact, indicating a lower influence of term-time forcing while retaining a similar mean basic reproductive ratio *R*_0_ of 33 (see Fig 4C). The range of each normalized local seasonal amplitude is shown in S3 Fig. Although the fit is not perfect, this model improves on the global formulation in capturing the modulation of epidemiologically relevant contact rates during WWII.

Previous analyses have suggested that varying patterns of transmission (other than seasonality) may influence the large dynamic shifts observed in these data. However, interestingly, our results show that a constant mean transmission rate (*R*_0_ = 29) modulated by a fixed pattern of seasonal variation mirroring the school-term predicts the majority of the data even as the attractor is hit by large internal and external perturbations. This is in contrast to the previous analysis of long-term data from New York city which concluded that a model with secular changes in transmission patterns was required to explain the data [12]. However, different dynamical regimes (limit cycles versus the edge of chaos) between the US and UK [5] may be a contributing factor.

### Lyapunov exponents

**Fig 5:**
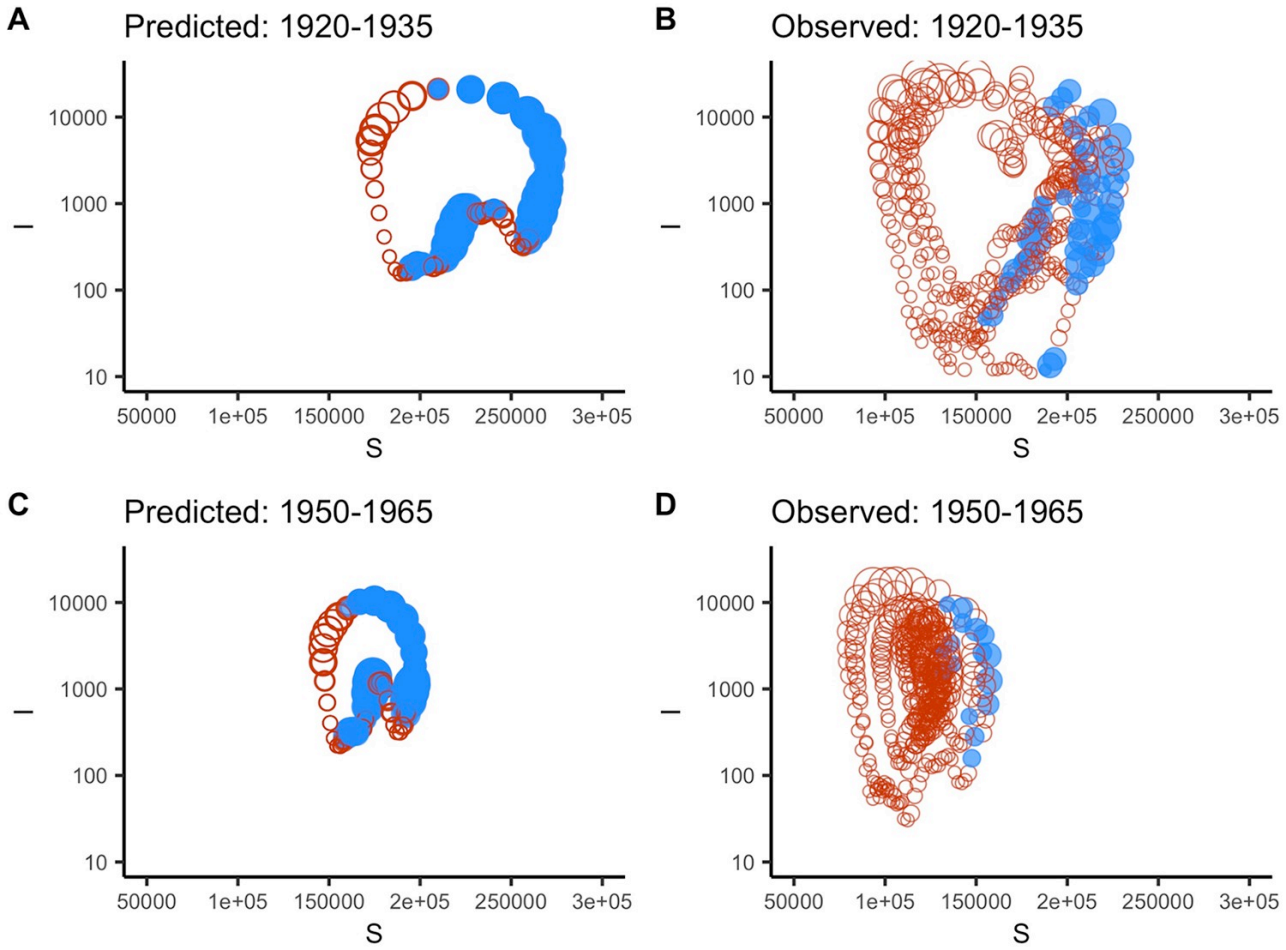
A comparison of predicted against observed measles Lyapunov exponents for two biennial attractors. A) Local Lyapunov exponents (LLEs) across the 1920–1935 biennial attractor predicted for mean birth rates. The filled blue circles indicate positive values with red indicating negative values. The size of the circle corresponds to the absolute value of the exponent. B) The observed LLEs across the 1920–1935 range using the true birth rates. C) Predicted LLE for the 1950–1965 biennial attractor using mean birth rates. D) Observed LLEs for the 1950–1965 data using the true birth rates.

To further quantify the stability of the system across 1897 to 1991, we calculated both local and global Lyapunov exponents [3]. From the fitted model, we calculated the dominant Lyapunov exponent, and found no evidence of chaotic dynamics, despite multiple dynamical jumps between the attractors (LE = -0.04, range: -0.28–0.11, Fig 5). This is again in contrast with pre-vaccination US measles, where chaotic (LE > 0) dynamics dominated [5]. In terms of local dynamics, the LLEs point to stability around two biannual attractors (1920–1935 and 1950–1965, Fig 5). However, LLEs in this context may be more qualitative, as we are comparing mean birth rates in an era (Fig 5A & 5C) against the time-varying, true birth rates (Fig 5B & 5D).

## Discussion

A key question in nonlinear ecological dynamics is how a system responds to external changes. Examining measles in London using a stochastic SEIR model, we were able to quantify the impact of external perturbations on the internal clockwork across nearly a century of data. We found strong agreement between the data and the fitted, forward-simulated model, indicating that both temporal dynamics, such as epidemic shape and size, and bifurcations can be accurately predicted. Assessing the stability of the London attractor using Lyapunov exponents, we found remarkable stability (LE < 0) across the time series, with most of the phase plane being highly dissipative (LLEs < 0), thereby accounting for the absence of long-term divergence even in the presence of stochasticity. Additionally, we show that a temporally stable seasonal transmission function is sufficient to reproduce the empirical dynamics despite any possible socio-economic changes during the last century.

Although sudden changes in periodicity may appear unpredictable, we show that knowledge of internal and external events, such as changes in birth rates and sudden population changes, is enough to produce accurate forecasts of periodicity and outbreak dynamics. By explicitly incorporating the reduction in contact rates associated with the key demographic perturbations in 20th century London, such as the 1918 pandemic and WWII evacuation, we demonstrate how subtle changes in contact rates, rather than more obvious changes such as a birth pulse, can lead to bifurcations and other changes in the dynamics of this nonlinear system (see also [5]). Our estimated contact function, particularly the finer-scale analysis during the WWII evacuation of school-age children, provides additional evidence for the role of schools in driving measles dynamics across multiple temporal scales [5,17,24]. Additionally, while estimated in a different framework, the contact pattern is similar to that previously estimated with the discrete time TSIR model using a subset of the data studied here [17,18]. Combining mortality and incidence data has additionally allowed us to extend the well-studied London time series and further our ability to test ecological theory with data.

Our analysis both contrasts with and complements the work of Hempel and Earn [12], who showed that understanding New York measles dynamics requires consideration of non-stationarities in the underlying nonlinear clockwork. Our London analysis, in contrast, suggests that changes in the dynamics resulted from pulsed perturbations and secular changes in susceptible recruitment rates. The identification of a single seasonality function around a constant *R*_0_ (with the exception of WWII) for the entire time series, given the scale of the changes observed in population size and schooling dynamics, provides a compelling case for the utility of considering simple mechanistic models when studying predictability of long-term nonlinear dynamics. A crucial difference between the two settings may be as pointed out by [5]: the US measles attractor appears to be more sensitive to small changes in seasonal forcing than that of the UK.

The discrepancy between the globally fitted forward simulation and the data during the WWII era (Fig 4) is intriguing. The lower amplitude seasonal pattern observed when estimated from the 1940–1946 period alone indicates that school-term forcing played less of a role during this time. This change likely reflects the city-wide evacuation. Given the age-structured nature of the exodus, it is perhaps not surprising that the well-mixed SEIR model could not predict these anomalous dynamics when (largely) trained on the other 88 years of data. Furthermore, the reliability and accuracy of demographic and incidence reporting during WWII is questionable and may have biased our inference framework locally, or even resulted in a mismatched attractor [25,26]. Given that measles cases became notifiable in 1940, initial reporting may have been less accurate [27]. Additionally, due to the evacuation, the Registrar General provides an explanatory statement in their September 9th, 1939 weekly report stating that “owing to the partial evacuation of populations from London …the weekly birth and death rates cannot be calculated with accuracy …the estimated populations at the middle of 1939 given in Table 1, no longer correspond even approximately with the deaths in that table”. Lastly, the inability to find London-specific vaccine data, and therefore using the population scaled country-level data, may have influenced our ability to drive the model with accurate susceptibility rates during the vaccine era. An additional complication in the vaccine data is the switch to the MMR vaccine in 1988, where a number of children were vaccinated twice [28]. However, varying the vaccine efficacy from 90% to 99% from 1988 on produced very little qualitative difference, likely due to the already very low incidence by this point.

Despite these limitations, we have nearly doubled the London measles analysis to include multiple transitions that have not previously been considered in tandem. Using a simple fitted model combined with an estimated CFR, we have shown how robust the nonlinear dynamics produced by the SEIR family of models can be despite multiple perturbations to the system that impact nearly every compartment of the model. Further work on predictability should continue to seek long time series in systems that experience critical dynamic shifts to further test the utility of applying ecological consumer-resource theory in the context of infectious disease dynamics. Finally, given the recent resurgence of measles due to vaccine hesitancy, our study lends itself to the public health importance of understanding the nonlinear dynamics of endemic transmission.

## Materials and methods

### Data

We analyzed weekly measles incidence and measles mortality reports in London from 1897 to 1991. In addition to the previously analyzed and published incidence data from 1944–1964 [5], and mortality data from 1904–1915 and 1922–1932 [4], we extended both the incidence (now 1940–1991) and mortality (1897–1940) time series to produce a continuous sequence of monitoring data for the entire period comprising 4,877 weekly data points across the 94 years. Official mortality and incidence notifications (measles cases became notifiable in 1940) were digitized from the Registrar General’s Weekly Reports [25]; annual birth rates and population estimates were obtained from the Registrar General’s Annual Reports [29], while estimates of life expectancy were obtained from the Office of National Statistics [30]. We used country-level averaged vaccination rates in scaling susceptible recruitment rates.

Due to administrative boundary changes, the population of Inner London changed from 4,449,040 in 1897 to 2,345,500 in 1991. During this time annual birth numbers varied greatly from 131,000 to 39,000, corresponding to crude birthrates of 30 to 12 (see Fig 1). These data span multiple historic events which acted as external perturbations, including World War One (WWI—1914–1918), World War Two (WWII—1939–1945), the 1918 influenza pandemic, and the broad-age vaccination pulse in 1968 (44,600 vaccine doses administered in 1968) during the roll-out of mass vaccination. The 1918 Influenza Pandemic (June 29th, 1918 to May 10th, 1919 in London) [31] resulted in 228,000 deaths in Britain, but more importantly for epidemic dynamics, resulted in both the closure of primary schools as well as other foci of high contact such as theaters [32,33]. Contact rates may have also been impacted during WWII as Operation Pied Piper (September 1st, 1939 to September 2nd, 1945) led to the evacuation of over a million civilians, primarily children, from London [6,34].

### Methods

Between 1940 and 1947, both measles mortality and case data were available. However, due to the small number of measles deaths in this period (less than 200 notified deaths versus over 100,000 reported cases), we used the case data from 1940 onward to capture the endemic pre-vaccination dynamics. Given that we were only interested in estimating a single set of initial conditions in 1897, we used iterated filtering methods in which parameters of interest take random walks with a fixed standard deviation to maximize the likelihood [24,35–37]. A discussion of the method and implementation can be found in S1 Text and in the following references [20,38]. To fully explore the large parameter space, we produced 1,000 different samples, each with 50 iterations and 2,000 particles. Once parameters were estimated, we stochastically calculated the likelihood ten times per estimate.

For the entire time series, we used a simple well-mixed Susceptible-Exposed-Infected-Recovered (SEIR) model with seasonal forcing in transmission (see S1 Text). Similar to [35], we used a stochastic framework with both demographic (epidemic birth-death) stochasticity and white noise environmental stochasticity in the force of infection. To examine whether a year-invariant pattern of transmission, *R*_0_, could capture the dynamic eras across time, we modeled the seasonally forced transmission rate using a spline with six degrees-of-freedom. The shape of seasonality is generally thought of as driven by changing contact patterns during opening and closing of schools. We estimated a single seasonal forcing function except for during the 1918 pandemic (discussed below), where transmission is modulated at a constant rate. We also included an additional parameter, the cohort effect, to capture the possibility that more children may effectively enter the susceptible class at the start of the school year (i.e. an annual susceptible recruitment pulse [39]). To account for potential under-reporting, 50% of measles cases were reported, in line with previous analyses [35] and full-reporting for measles mortality. For both the mortality and incidence notifications, we allowed for error in the reporting via dispersion parameters, per [35].

To test the model’s ability to capture multiple transients across the time series, we specified three main elaborations to the standard SEIR model. First, we explicitly incorporated the reduction in contact due to the 1918 pandemic when schools and theaters were closed as a public health measure. If a reduction in contact is inferred, the pattern of seasonality does not change, simply the magnitude during the pandemic year decreases. Second, we estimated a case fatality rate (CFR) [40] from 1897 to 1940 to make a bridge from mortality to incidence data. To preserve local variability in the measles fatality rate, the CFR was inferred using a Gaussian Process regression between cumulative deaths and cumulative births. Although the estimated CFR impacts the magnitude of predicted epidemics, it does not impact the periodicity and was thus used as a covariate (similar to the population data) in the analysis. Finally, we subtracted vaccines from the susceptible compartment assuming 90% efficacy [28] during the 1968 roll-out, since the vaccine was initially used primarily as a catch-up campaign (i.e. immunization of a broad age range) during this period [41,42]. Once parameters are estimated, we can forward simulate the fitted model to compare against the data.

To explore the interaction between stochastic and nonlinear dynamics in recurrent epidemics, we evaluated both the global and local Lyapunov exponents (LEs and LLEs, respectively). While LEs give a measure of overall sensitivity to initial conditions and overall dissipativeness (i.e. the ability of a system to return to a steady state) across the measles attractor, the LLEs show where along the attractor noise and/or perturbation are likely to lead to divergent epidemic trajectories (LLE > 0), and where the nonlinear clock-work will contract epidemics onto similar trajectories (LLE < 0) [9]. To facilitate ease of calculation of the LE and LLEs, we used the discrete-time time series SIR (TSIR) approximation of the SEIR model with time-steps scaled to be biweekly. Predicted LLEs were obtained from calculating the deterministic skeleton of the fitted TSIR model. The R package tsiR [18] has been updated with functionality to calculate both local and global Lyapunov exponents.

Additionally, we performed a wavelet spectra analysis to quantify measles periodicity throughout the time series [13,43]. This descriptive analysis yields insight into whether the observed dynamics (as well as the fitted model forward simulations) are annual, biennial, or three-plus year cycles at each time step. Measuring the periodicity over time for multiple stochastic simulations from the fitted model allows for a probabilistic comparison between the observed and predicted dynamics.

All analysis was performed using the R programming language [44] with the ggplot2 [45], cowplot [46], tsiR [18,47], Rwave [48], and pomp [38,49] packages.

## Supporting information

S1 Text Description of the model, fitting processes, and fitted parameters. (DOCX)

S1 Table Parameters from the stochastic SEIR model. For each parameter, we denote a short description, whether the parameter is time-varying or constant, if it is fixed or estimated, and the value with 95% confidence intervals when applicable.
(DOCX)

S1 Fig Profile log likelihood calculation of the reduction in contact due to the 1918 pandemic year. The maximum likelihood estimate is 38% reduction.
(TIF)

S2 Fig Inferred case fatality ratio for measles between 1897–1940. (TIF)

S3 Fig The inferred local normalized seasonality ranges for six-year windows throughout the time series. The red point shows the inferred WWII range, whereas the black star points to the overall inferred range. The box plot then shows the ranges across the other local eras. The WWII pattern has the smallest range across all the local inferences, indicating a lower presence of school-term forcing during the evacuation.
(TIF)

S4 Fig Local power spectra for the death and case data. The black outlines denote significance levels with red referring to dominant periodicity and blue low levels of inferred periodicity.
(TIF)

S1 Data The demography data analyzed as a csv file. (CSV)

S2 Data The incidence and mortality data analyzed as a csv file. (CSV)
