# Supplementary material for: Long-term dynamics of measles in London: Titrating the impact of wars, the 1918 pandemic, and vaccination
Source: PLoS Comput Biol. 2019 Sep 12;15(9):e1007305. doi: 10.1371/journal.pcbi.1007305 (PMC6742223; doi:10.1371/journal.pcbi.1007305)
Supplement: S1 Text — (DOCX) [file pcbi.1007305.s001.docx]

**Supplement: Long term dynamics of measles in London: titrating the impact of wars, the 1918 pandemic, and vaccination**

*SEIR model description*

In this section, we describe our fitted stochastic SEIR model and parameters. For simplicity, we show the deterministic equations. Note however, that each transition is modeled stochastically as Euler-multinomial transitions between states. Each time step in the model was set to be $\Delta t=1$ day.

$$\frac{dS}{dt}=Births-\lambda_{t}({I+\iota)}^{\alpha}-\mu_{t}S-Vaccination$$

$$\frac{dE}{dt}=\lambda_{t}({I+\iota)}^{\alpha}-\sigma E-\mu_{t}E$$

$$\frac{dI}{dt}=\sigma E-\gamma I-\mu_{t}I$$

$$\frac{dR}{dt}=\gamma I-\mu_{t}R$$

Here, $\alpha$ is a mixing parameter, $\gamma$ and $\sigma$ the recovery and infection rate, respectively, and $\mu_{t}$ is the death rate, assumed a known covariate from life expectancy data. Births are allowed to vary seasonally based on the cohort parameter, $c$, allowing for a greater influx of susceptible individuals during the first week of school, assumed to be the first week of September. In this week, the birthrate changes from

$$birthrate=(1-c)*B_{t}$$

To

$$birthrate=c*B / \Delta t+ (1-c)*B_{t}$$

Where $B_{t}$ refers to the true birth rate from the data. We can further add demographic stochasticity into the model via:

$$Births \sim pois(birthrate*\Delta t)$$

$$Vaccination \sim pois(vaccination\_rate*V_{e}*\Delta t)$$

Where the vaccination rate is also a time dependent covariate assumed known from the data, and $V_{e}$ is the assumed vaccine efficacy ( = 90%). Extra demographic stochasticity was further incorporated into the force of infection:

$$\lambda_{t}= \beta_{t}SN^{-1}W$$

Where $\beta_{t}$ is the estimated seasonally repeating contact function with six degrees of freedom. To account for potential closures due to the 1918 influenza pandemic, we allow $\beta_{t}$ to be modulated during the 1918 pandemic year (e.g. be reduced by x%). Contact $\beta_{t}$ can be related to the crucial transmissibility parameter, $R_{0}$, by the approximation $R_{0}=$mean ($\beta_{t}) / \gamma$. Lastly, $W$refers to a gamma white noise parameter with standard deviation $\sigma_{SE}$ as per (*1*). Additionally, a set of initial conditions for each state is further estimated. This model can be simulated using guess parameters, however we proceed via the fitting procedure described below. Note that in our analysis, the fit shown in Figure 2 is the re simulated model constructed with the estimated parameters started using the single set of initial conditions.

*Fitting Method*

To configure our stochastic epidemic model against our weekly incidence and mortality data (as well as the known covariates such as births and vaccination), we relate cases in the model (denoted here as C) with the weekly case data (denoted here as *cases*) by summing the number $\gamma I$ in a seven-day period. We model deaths (D) by $\phi$C, where $\phi$ is the case fatality rate. These model states (C and D) can be further calibrated against the data (*cases* and *deaths*) by our choice of likelihood function. Based on the work of (*1*), we assume an over-dispersed binomial model with both the potential for under-reporting ($\rho_{C}$ or $\rho_{D})$and measurement error ($\psi_{C}$ or $\psi_{D})$.

$$P\left[ cases \right|C]=\Phi\left( cases+0.5, \rho_{C}C, \rho\left( 1-\rho_{C} \right)C+ \psi_{C}^{2}{\rho_{C}}^{2}C^{2} \right)-$$

$$\Phi(cases- 0.5, \rho_{C}C, \rho_{C}\left( 1-\rho_{C} \right)C+ \psi_{C}^{2}\rho_{C}C^{2})$$

Where $\Phi$ refers to the cumulative normal distribution. The likelihood function for *deaths* is the same, just replacing *cases* with *deaths*, and therefore C with D. When *cases* or *deaths* are zero this function relaxes to:

$$P\left[ cases \right|C]=\Phi\left( cases+0.5, \rho_{C}C, \rho\left( 1-\rho_{C} \right)C+ \psi_{C}^{2}{\rho_{C}}^{2}C^{2} \right)$$

To estimate parameters throughout the full time series, and therefore combine the case and death data, we switch likelihood functions based on era, such that we only use the *cases* function after 1940. This allows parameters to be estimated using the whole era as the underlying *S*, *E*, *I*, and *R* states remain unaffected by the choice of likelihood function.

Using the above model and likelihood formulation, we estimated parameters using the Iterated Filter algorithm implemented in the pomp R package (*2*, *3*). A brief qualitative overview of the algorithm follows, although a more exhaustive theoretical background can be found in (*4*, *5*).

The central idea of the algorithm is to allow parameters of interest (e.g. $\beta_{t}$) to take random walks with a fixed standard deviation (set here to be 0.02 in exponentially transformed space). Each iteration, stochastic trajectories are simulated from these random walks, and compared against the data using the above likelihood functions. To obtain convergence and to smooth the likelihood surface (to avoid local minima / maxima), we reduce the variance by 50% over the course of our 50 iterations. Repeating this process for a number of starting parameter sets (here we use 1,000), allows us to explore the parameter space.

Once the iterations are complete, we are left with parameters and their associated likelihood estimates (computed again from the above function). The maximum likelihood estimate (MLE) is our model estimate. We can evaluate our MLE by re simulating the model (described above) using these new parameters. Notably, the simulations produced by the fitted model (as shown in Figures 2-4) occur after the fitting process itself.

*Estimated parameters*

Generally, the estimated parameters in our analysis agree with previous inferences. The initial conditions (fitted just once in 1897) are similar to those estimated by (*1*) using the same framework, as well as those estimated by (*6*) using the TSIR model. Multiple of our estimates fall below those in (*1*) the immigration, white noise, cohort, and case reporting dispersion parameters (their estimates were 2.9, 0.09, 0.56, and 0.12, respectively for London; 1950-1965). However, this is likely due to carrying our inference throughout the vaccine era, when the number of infected imports per year likely decreased, as well as the number of school-age children susceptible. Our estimated $R_{0}$ of 29 is above the typical range of 12-18 given by (*7*), but similar to the London estimate ($R_{0}$ = 30) obtained by (*8*), and below the estimated $R_{0}$ = 57, also from London, from (*1*) using the same method

A full table of each parameter, whether it is time-varying or not, whether it is estimated or fixed, and the value with 95% confidence intervals (calculated by the chi-square approximation of the likelihood ratio test), if applicable, follows.

| $\theta$ | Description | Time-varying | Estimated | Value | CI (95%) |
| --- | --- | --- | --- | --- | --- |
| $\phi_{t}$ | Case fatality rate | 1897 - 1940 | Y | 0.0160 | 0. 0157 – 0.0161 |
| $flu$ | 1918 pandemic reduction in contact | 1918 - 1919 | Y | 38% | 17% - 70% |
| $1/\sigma$ | Latent period | N | N | 8 days | NA |
| $1/\gamma$ | Infectious period | N | N | 5 days | NA |
| $R_{0}$ | Basic reproductive number | Seasonally | Y | 29 | See figure 2 |
| $\alpha$ | Heterogeneity parameter | N | N | 0.975 | NA |
| $\iota$ | Infectious import rate | N | Y | 0.31 | 0.30 – 0.31 |
| $\rho_{C}$ | Reporting rate (cases) | N | N | 50% | NA |
| $\rho_{D}$ | Reporting rate (deaths) | N | N | 100% | NA |
| $\sigma_{SE}$ | Multiplicative white noise | N | Y | 9.6e-5 | 7.8e-5 - 2.9e-4 |
| $\psi_{C}$ | Reporting dispersion (cases) | N | Y | 6e-4 | 4.9e-4 – 9.1e-4 |
| $\psi_{D}$ | Reporting dispersion (deaths) | N | Y | 2e-5 | 1.4e-5 - 3.1e-5 |
| $c$ | Cohort effect | N | Y | 2e-3 | 1.8e-3 - 3.1e-3 |
| $S(0)$ | Initial proportion susceptible | N | Y | 0.0311 | 0.031 – 0.317 |
| $E(0)$ | Initial proportion exposed | N | Y | 5e-4 | 5e-5 – 5.1e-5 |
| $I(0)$ | Initial proportion initials | N | Y | 2.66e-4 | 2.65e-4 – 2.69e-4 |
| $R(0)$ | Initial proportion recovered | N | Y | 0.968 | 0.967 – 0.968 |
| $1/\mu_{t}$ | Life expectancy | Globally | N | 63 years | 55 – 71 |
| $V_{e}$ | Vaccination efficacy | N | N | 90% | NA |

*Table S1: Parameters from the stochastic SEIR model. For each parameter, we denote a short description, whether the parameter is time-varying or constant, if it is fixed or estimated, and the value with 95% confidence intervals when applicable.*

*Pandemic year reduction*

The likelihood profile for the 1918 reduction in contact is shown below. A strong peak is identified at 40%, notably the likelihood argues against a zero reduction.


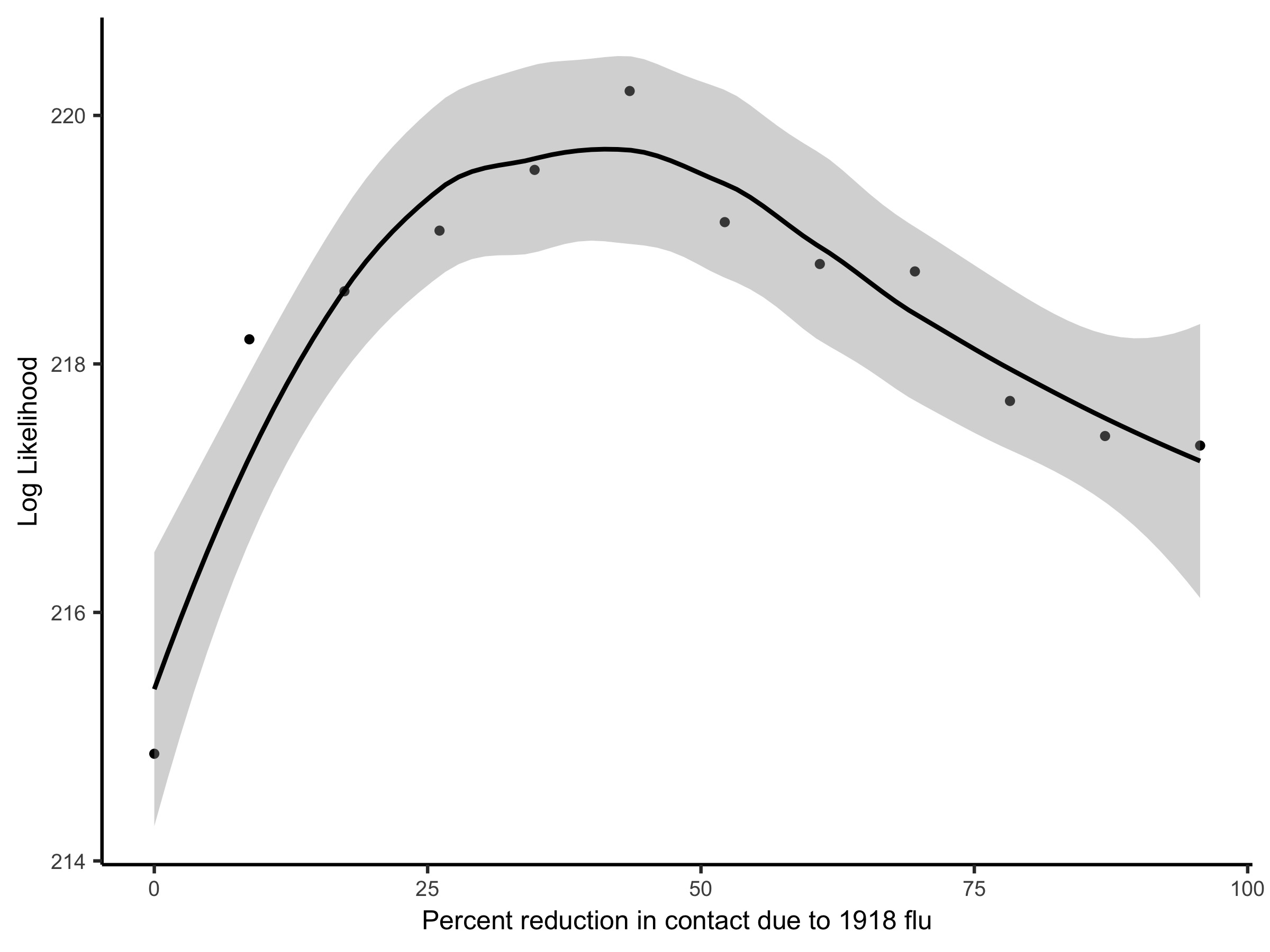


Figure S1: Profile log likelihood calculation of the reduction in contact due to the 1918 pandemic year. The maximum likelihood estimate is 38% reduction.

*Lyapunov Exponent Analysis*

The observed LLEs match the predicted exponents well, however there is tendency to overestimate negative values during 1950-1965. The fitted model may be biased towards negative values due to the seasonality being inferred from the entire time series, as opposed to the just the region of interest such as (*8*). The trajectories of the observed dynamics display more dispersion than the predicted, but that is to be expected when comparing true birth rates versus the simple average.

*Case fatality ratio*

The case fatality ratio was estimated using a Gaussian Process regression between cumulative deaths and cumulative births using the tsiR package (*9*) and is shown in Figure S2. Although using mortality data can provide insight into the underlying infectious disease dynamics (*10*, *11*), transitioning between death and case data presents the undesirable problem of having to estimate a time-varying CFR. While the assumption of using births and deaths to infer CFR yielded a realistic fit, it must be done *a proiri* to the rest of the model fitting.


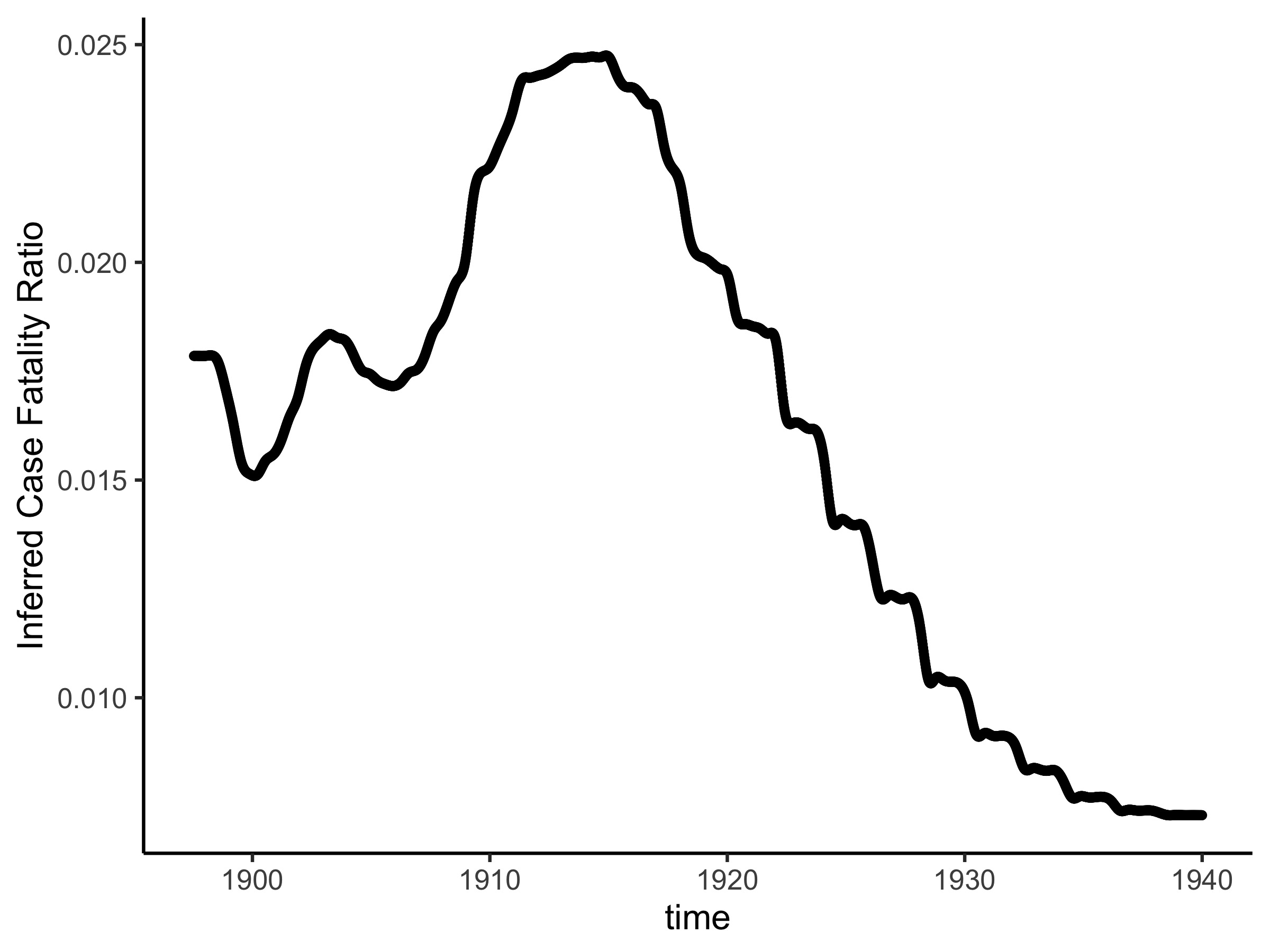


Figure S2: Inferred case fatality ratio for measles between 1897-1940.

*Local inference*

In order to test whether the WWII time-period had an appreciable change in seasonality (i.e. one that could not be detected in the 1897-1991 analysis), we fit the SEIR model to local six-year sections of data. Initial conditions across each time period were very similar, with the main difference being the range of seasonality values once normalized. We found that the 1940-1946 region had the smallest range, and was the flattest seasonality.


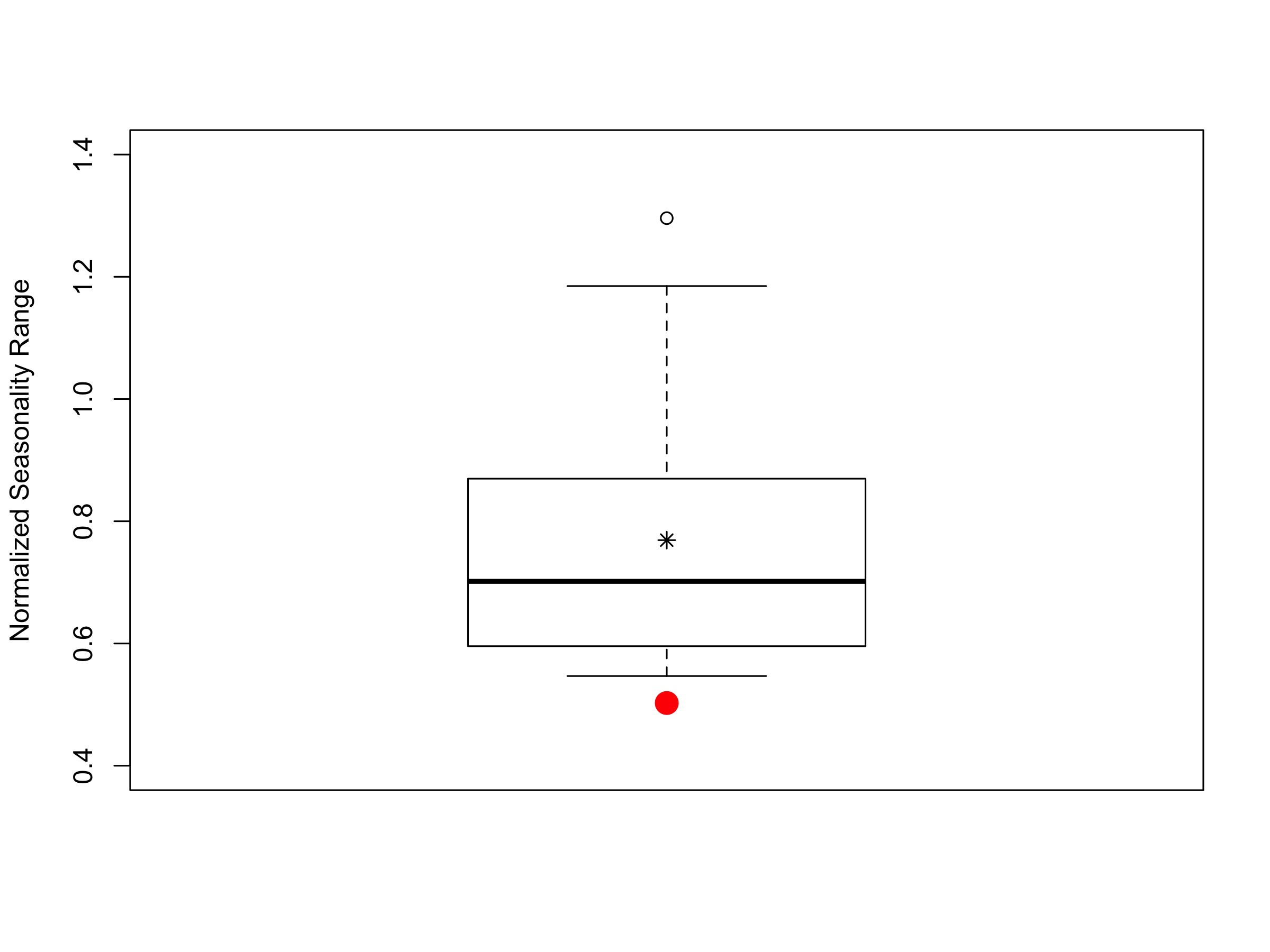


Figure S3: The inferred local normalized seasonality ranges for six-year windows throughout the time series. The red point shows the inferred WWII range, whereas the black star points to the overall inferred range. The box plot then shows the ranges across the other local eras. The WWII pattern has the smallest range across all the local inferences, indicating a lower presence of school-term forcing during the evacuation.

*Wavelets*

In order to examine the local periodicity of the death and case measles data, a wavelet analysis was performed and shown below. The red regions outlined in black indicate significant periodicities. We used the Rwave (*12*) package for this analysis, using the ‘cwt’ and ‘Mod’ functions. To quantify dominant frequencies in both the data and simulated fitted model, we extract the greater periodic signal at each time step. For example, in 1905, the death data shows a dominant annual signature, so we classify that as a one-year-cycle at that time point.


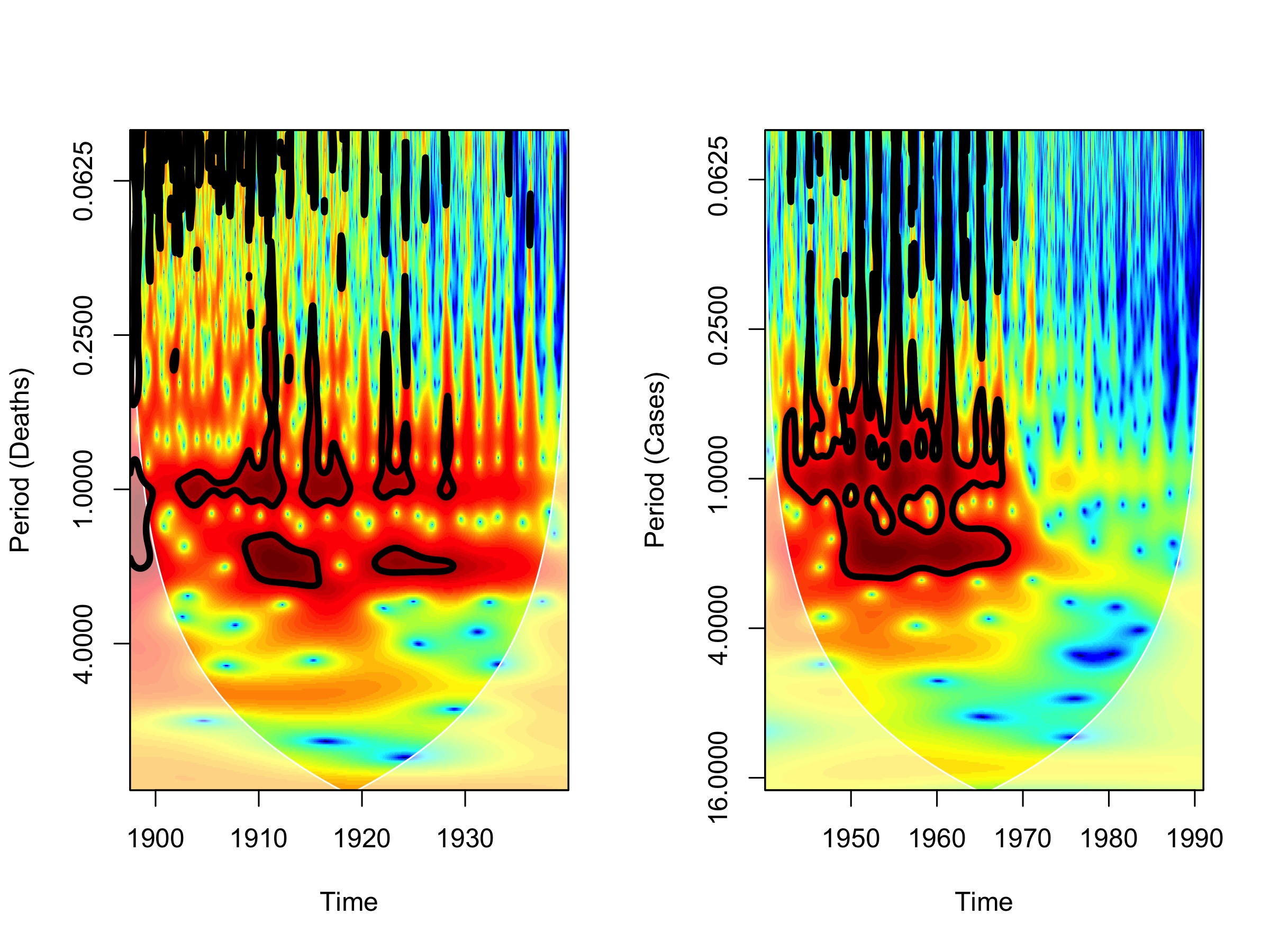


Figure S4: Local power spectra for the death and case data. The black outlines denote significance levels with red referring to dominant periodicity and blue low levels of inferred periodicity.

1. D. He, E. L. Ionides, A. A. King, Plug-and-play inference for disease dynamics: measles in large and small populations as a case study. *J. R. Soc. Interface*. **7**, 271–283 (2010).

2. A. A. King, D. Nguyen, E. L. Ionides, Statistical Inference for Partially Observed Markov Processes via the *R* Package **pomp**. *J. Stat. Softw.* **69**, 1–43 (2016).

3. A. A. King *et al.*, pomp: Statistical Inference for Partially Observed Markov Processes (2015), (available at https://cran.r-project.org/web/packages/pomp/index.html).

4. C. Bretó, D. He, E. L. Ionides, A. A. King, TIME SERIES ANALYSIS VIA MECHANISTIC MODELS. *Ann. Appl. Stat.* **3**, 319–348 (2009).

5. E. L. Ionides, C. Bretó, A. A. King, Inference for nonlinear dynamical systems. *Proc. Natl. Acad. Sci. U. S. A.* **103**, 18438–18443 (2006).

6. O. N. Bjørnstad, B. F. Finkenstädt, B. T. Grenfell, Dynamics of measles epidemics: Estimating scaling of transmission rates using a Time series SIR model. *Ecol. Monogr.* **72**, 169–184 (2002).

7. R. M. Anderson, R. M. May, *Infectious diseases of humans: Dynamics and control.* (Oxford: Oxford University Press, 1992; http://onlinelibrary.wiley.com/doi/10.1002/hep.1840150131/abstract), vol. 15.

8. B. T. Grenfell, O. N. Bjørnstad, B. F. Finkenstädt, Dynamics of measles epidemics: scaling noise, determinism, and predictability with the tsir model. *Ecol. Monogr.* **72**, 185–202 (2002).

9. A. D. Becker, B. T. Grenfell, tsiR: An R package for time-series Susceptible-Infected-Recovered models of epidemics. *PLoS One*. **12**, e0185528 (2017).

10. N. B. Mantilla-Beniers, O. N. Bjørnstad, B. T. Grenfell, P. Rohani, Decreasing stochasticity through enhanced seasonality in measles epidemics. *J. R. Soc. Interface*. **7**, 727–39 (2010).

11. A. A. King, E. L. Ionides, M. Pascual, M. J. Bouma, Inapparent infections and cholera dynamics. *Nature*. **454**, 877–880 (2008).

12. J. M. L. Rene Carmona, Bruno Torresani, Brandon Whitcher, Andrea Wang, Wen-Liang Hwang, CRAN - Package Rwave, (available at https://cran.r-project.org/web/packages/Rwave/index.html).
