# Supplementary material for: Long-term dynamics of measles in London: Titrating the impact of wars, the 1918 pandemic, and vaccination
Source: PLoS Comput Biol. 2019 Sep 12;15(9):e1007305. doi: 10.1371/journal.pcbi.1007305 (PMC6742223; doi:10.1371/journal.pcbi.1007305)
Supplement: S1 Table — For each parameter, we denote a short description, whether the parameter is time-varying or constant, if it is fixed or estimated, and the value with 95% confidence intervals when applicable. (DOCX) [file pcbi.1007305.s002.docx]

| $\theta$ | Description | Time-varying | Estimated | Value | CI (95%) |
| --- | --- | --- | --- | --- | --- |
| $\phi_{t}$ | Case fatality rate | 1897 - 1940 | Y | 0.0160 | 0. 0157 – 0.0161 |
| $flu$ | 1918 pandemic reduction in contact | 1918 - 1919 | Y | 38% | 17% - 70% |
| $1/\sigma$ | Latent period | N | N | 8 days | NA |
| $1/\gamma$ | Infectious period | N | N | 5 days | NA |
| $R_{0}$ | Basic reproductive number | Seasonally | Y | 29 | See figure 2 |
| $\alpha$ | Heterogeneity parameter | N | N | 0.975 | NA |
| $\iota$ | Infectious import rate | N | Y | 0.31 | 0.30 – 0.31 |
| $\rho_{C}$ | Reporting rate (cases) | N | N | 50% | NA |
| $\rho_{D}$ | Reporting rate (deaths) | N | N | 100% | NA |
| $\sigma_{SE}$ | Multiplicative white noise | N | Y | 9.6e-5 | 7.8e-5 - 2.9e-4 |
| $\psi_{C}$ | Reporting dispersion (cases) | N | Y | 6e-4 | 4.9e-4 – 9.1e-4 |
| $\psi_{D}$ | Reporting dispersion (deaths) | N | Y | 2e-5 | 1.4e-5 - 3.1e-5 |
| $c$ | Cohort effect | N | Y | 2e-3 | 1.8e-3 - 3.1e-3 |
| $S(0)$ | Initial proportion susceptible | N | Y | 0.0311 | 0.031 – 0.317 |
| $E(0)$ | Initial proportion exposed | N | Y | 5e-4 | 5e-5 – 5.1e-5 |
| $I(0)$ | Initial proportion initials | N | Y | 2.66e-4 | 2.65e-4 – 2.69e-4 |
| $R(0)$ | Initial proportion recovered | N | Y | 0.968 | 0.967 – 0.968 |
| $1/\mu_{t}$ | Life expectancy | Globally | N | 63 years | 55 – 71 |
| $V_{e}$ | Vaccination efficacy | N | N | 90% | NA |
