# Supplementary figures and images for: Long-term dynamics of measles in London: Titrating the impact of wars, the 1918 pandemic, and vaccination
Source: PLoS Comput Biol. 2019 Sep 12;15(9):e1007305. doi: 10.1371/journal.pcbi.1007305 (PMC6742223; doi:10.1371/journal.pcbi.1007305)

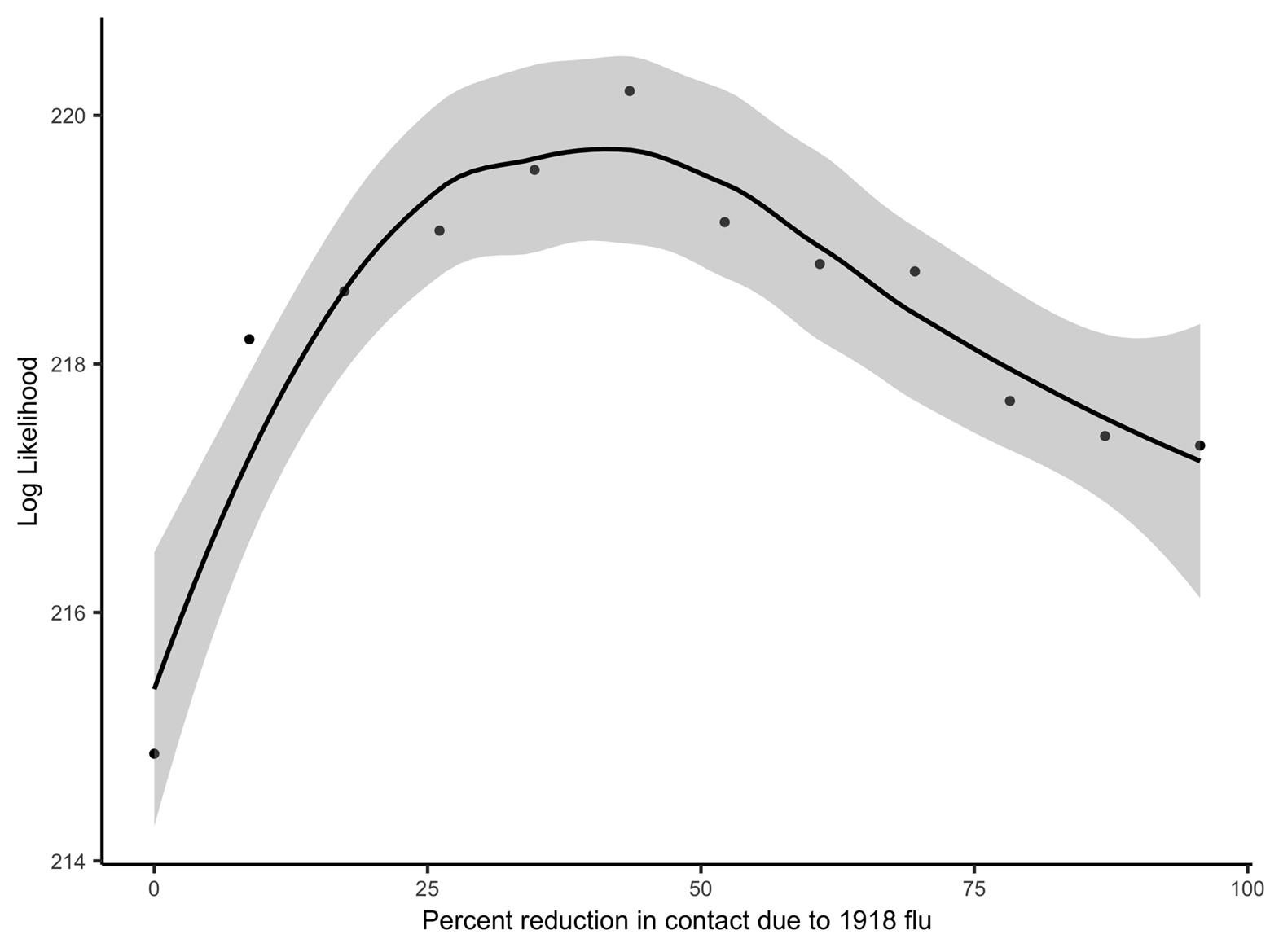

Supplement: S1 Fig — The maximum likelihood estimate is 38% reduction. (TIF) [file pcbi.1007305.s003.tif]

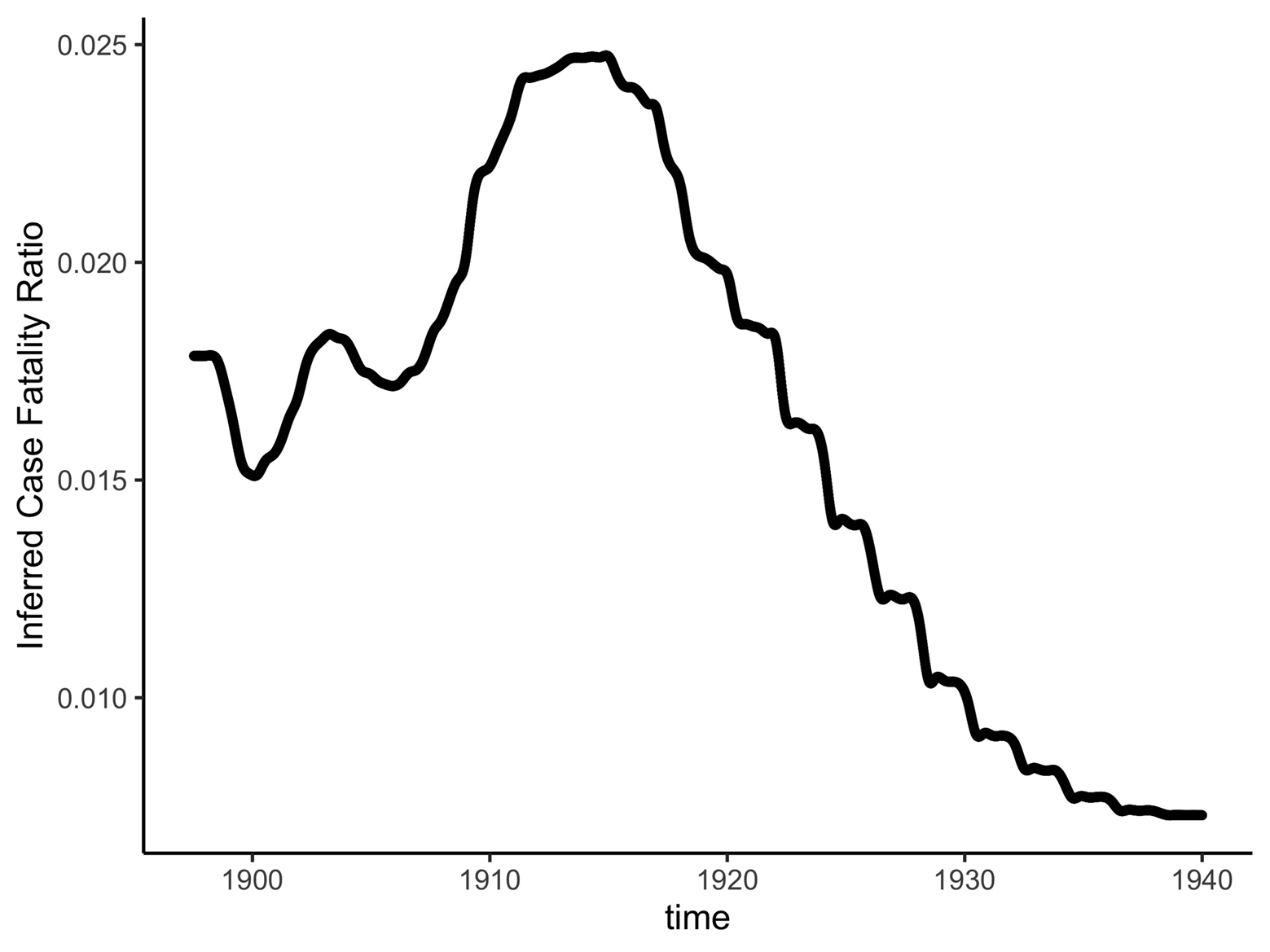

Supplement: S2 Fig — (TIF) [file pcbi.1007305.s004.tif]

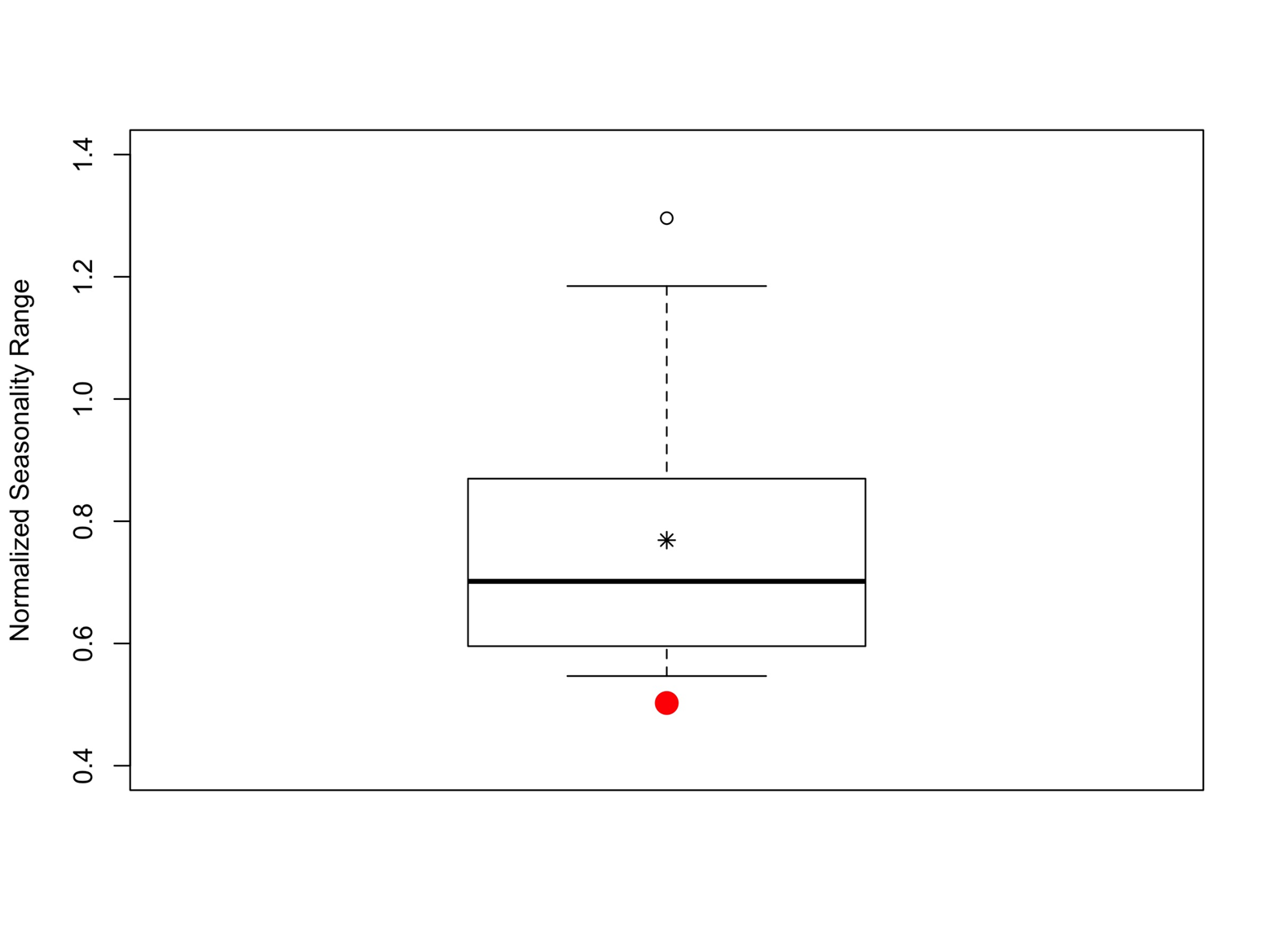

Supplement: S3 Fig — The red point shows the inferred WWII range, whereas the black star points to the overall inferred range. The box plot then shows the ranges across the other local eras. The WWII pattern has the smallest range across all the local inferences, indicating a lower presence of school-term forcing during the evacuation. (TIF) [file pcbi.1007305.s005.tif]

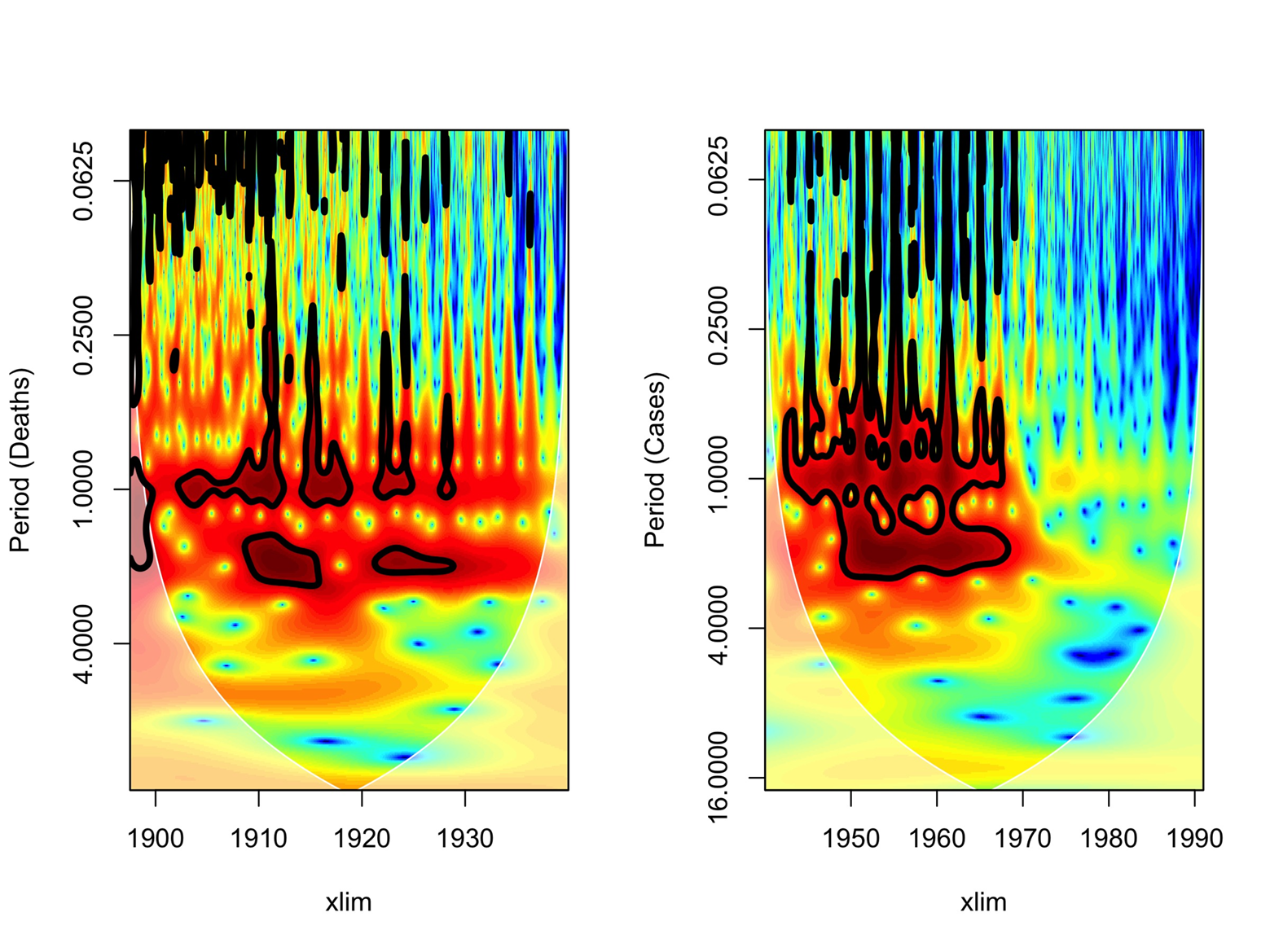

Supplement: S4 Fig — The black outlines denote significance levels with red referring to dominant periodicity and blue low levels of inferred periodicity. (TIF) [file pcbi.1007305.s006.tif]
